# Supplementary material for: SKiM: accurately classifying metagenomic ONT reads in limited memory
Source: Bioinformatics. 2025 Sep 24;41(10):btaf537. doi: 10.1093/bioinformatics/btaf537 (PMC12502918; doi:10.1093/bioinformatics/btaf537)
Supplement: btaf537_Supplementary_Data [file btaf537_supplementary_data.zip › Supplementary_Information_SKiM.pdf]

Supplementary Information: SKiM

S1 Proof of Adaptive-Blocks Theorem

Lemma 1. *Given an arbitrary bit string B, Algorithm 1 encodes B in the fewest possible number of runs (run of zeros, run of ones, or uncompressed).*

Proof. By "greedy stays ahead."

We start by assuming that the maximum length of a run block is $2^{w-2}-1$ and an uncompressed block holds $w-1$ bits of the original string.

Let G be the list of runs (zeros, ones, or uncompressed) produced by running Algorithm 1 (the greedy algorithm) on B and let $\mathcal{O}$ be the runs of an optimal solution, that, by definition, encodes B in the fewest possible runs.

Considering the first x runs of a solution, our progress measure is how many bits of the original string B are encoded by these x runs. Let g_i be the ith progress measure from G, e.g., g_3 is how many bits of B are encoded by the first three runs. Likewise, let o_j is the jth progress measure of $\mathcal{O}$.

We want to prove that, for all k, $g_k \geq o_k$. In other words, that the first k runs of G encode at least as many bits as the first k runs of $\mathcal{O}$ for all values of k.

We assume that, w.l.o.g., runs of zeros or ones can be considered the same case, which we refer to as compressed runs. Also, for the simplicity of the proof, we assume that compressed runs will not overflow, although we could extend the proof to not have this assumption. Finally, we point out two key invariants of the greedy algorithm: First, assuming the first x runs encode the first ℓ bits of B, then adding the next run $(x+1)$ will always encode at least the first $\ell+w-1$ bits of B (adding an uncompressed run), where w is the word size. In other words, the greedy algorithm will never choose to encode less than $w-1$ bits in the next run. Second, if there is a sequence of the same bit, starting at position $\ell+1$, that extends beyond position $\ell+w-1$, then the greedy algorithm will choose to encode the next run as a compressed run that is as long as possible. More precisely, if the first bit that is opposite of $B[\ell+1]$, starting from position $\ell+1$, occurs at position n, and $n-1 > \ell+w-1$, the greedy algorithm will encode out to position $n-1$ using a compressed run next.

We will now prove the original statement, by induction:

Base case. There are two options for the optimal solution on the first run. First, the optimal solution could use an uncompressed run and encode the first $w-1$ bits of B. As stated by the first invariant, the greedy algorithm will always encode at least this many bits from the starting position. Otherwise, the optimal solution uses a compressed run out to some position m. If $m \leq w-1$, then the greedy algorithm will still encode at least as many bits by the first invariant. If $m > w-1$, then there must be some position, n, at which the first bit that is opposite of $B[0]$ occurs, noting that $m \leq n-1$. Because $m > w-1$, then $n-1 > w-1$ must be true, and the greedy algorithm would choose to encode out to position $n-1$ using a compressed run according to the second invariant. In all cases, $g_1 \geq o_1$.

Inductive Step. Assume that $g_k \geq o_k$. We want to prove that $g_{k+1} \geq o_{k+1}$. This case is very similar to the base case, with the difference being that the greedy algorithm *could* start with more of B encoded. If the optimal solution uses an uncompressed run, $o_{k+1} = o_k + w - 1$. From our first invariant, we know that $g_{k+1} \geq g_k + w - 1$, so $g_{k+1} \geq o_{k+1}$ is true. If the optimal solution uses a compressed run, its total encoding will be out to some position $o_{k+1} = m$. If $m \leq g_k + w - 1$, then $g_{k+1} \geq o_{k+1}$ is still true by the first invariant. If $m > g_k + w - 1$, then there must be some position, n, where, starting at position $o_k + 1$, the first bit opposite of $B[o_k+1]$ occurs, again noting that $m \leq n-1$. Because $m > g_k + w - 1$, then $n - 1 > g_k + w - 1$ must be true, and the greedy algorithm would choose to encode out to position $n-1$, starting from position g_k, using a compressed run according to the second invariant. In all cases, $g_{k+1} \geq o_{k+1}$.

Based on this proof by induction, we assert that for every run in the optimal solution, the greedy solution encodes at least as many bits from the original bit string B, maybe more, in the same number of runs. Therefore, by the end of the greedy encoding, the total number of runs required by the greedy algorithm must be less than or equal to the number of runs required by an optimal solution (or simply equal to, because $\mathcal{O}$ was assumed to be optimal). □

Theorem 1. *Let* Adaptive-Blocks(M) *be the total number of runs required to represent any bit matrix M, obtained by running Algorithm 1 on each of the rows of M. Then* Adaptive-Blocks(M) $\leq$ Naive-Runs(M) *for any bit matrix M.*

Proof. First, we assume that the maximum length of a run block is $2^{w-2}-1$ for both Adaptive-Blocks(M) and Naive-Runs(M) and an uncompressed block holds $w-1$ bits of the original string for Adaptive-Blocks(M). By Lemma 1, Algorithm 1 encodes an arbitrary bit string B in the fewest possible blocks. In other words, running Algorithm 1 on each row in M results in each row requiring the fewest number of possible blocks given the constraints. Therefore, by definition, each row j's ARLE representation requires a number of blocks less than or equal to j's NRLE representation. Since this can be said for every row in the matrix, we conclude that Adaptive-Blocks(M) $\leq$ Naive-Runs(M) for any bit matrix M. □

S2 Selection of k for SKiM

SKiM's database and classification methods were designed for short k-mers. Importantly, the number of possible k-mers decreases exponentially with k, which quickly decreases database size as k decreases. Therefore, we wanted SKiM to use the smallest possible k to keep database sizes small. Typically, however, shorter k-mers lead to misclassifications for the reasons listed in Section 2.2. Our selection of k-mer size balances these considerations.

Specifically, one benefit of a small value of k is with memory overhead per k-mer. As stated in Section 2.1, we store the database matrix M row-wise (per k-mer), where each row is a vector (that stores an ARLE). Each vector must store a pointer and a length value (since vectors do not need to be mutable after the database is created, we do not need to store a capacity value). Consequently, each k-mer row requires 16 bytes of overhead, assuming each pointer and length value is 8 bytes. As k decreases, the total size of this overhead decreases exponentially.

The other benefit is that, for our reordered database matrix M', Naive-Runs(M') (and consequently Adaptive-Blocks(M')) is likely to be smaller for smaller values of k. In other words, the total number of runs required to represent our database is smaller (See Section 2.1). This is true mainly because the dimensionality of the columns is exponentially less for smaller values of k. Intuitively, two reference assemblies may not share many 31-mers because there are many positions for sequences to diverge, but they may share more 15-mers.

As an example, SKiM's default parameters ($k = 15$, $s = 9$, $t = 2$) have approximately 63 million unique syncmers, or about 12% of all canonical 15-mers. Assuming all syncmers are present in the reference database (which is likely given the large reference sizes we target), the vector pointers and length values take up 1GB of space. SKiM's $k = 16$ parameters ($k = 16$, $s = 10$, $t = 2$) also have about 12% of all canonical 16-mers. However, this means that it has four times as many unique syncmers, resulting in 4GB of vector pointers and length values. The difference in classification accuracy, classification speed, and database size between these two references can be seen in Sections 3.2 and 3.3. Namely, there is a minor bump to classification accuracy on short reads from $k = 15$ to $k = 16$ (which becomes negligible as the reads get longer) and a +50% boost to classification speed, but the peak RSS almost doubles from 14.6GB to 26.5GB.

We considered using $k = 14$, and we found the classification accuracy decreases, but is manageable. However, decreasing k increases the runtime (decreases throughput) as well. We determined both negatives in combination were not an improvement over SKiM's default parameters.

The $k = 16$ SKiM database is also substantially outperformed in terms of classification accuracy on short read lengths by the parameters $k = 15$, $s = 13$, $t = 1$. These $k = 15$ parameters sub-sample to about 25% of all canonical 15-mers, and have a peak RSS on par with the $k = 16$ parameters. Despite the slower classification speed, the increased accuracy earlier in the read is arguably more important for our targeted application.

Overall, SKiM's default parameters (particularly $k = 15$) should work for up-to-date ONT workflows, including adaptive sampling. Despite this, we give the user the option to choose $k = 14$, $k = 15$, or $k = 16$ along with the sub-sampling parameters (s and t).

S3 Simulated Reads

We used Badread [1] to generate the three simulated datasets, mimicking the theoretical abundance of the Even dataset (i.e., the ZymoBIOMICS Microbial Community Standard, see Section 3.1). More specifically, the 8 bacterial species in the standard each had 12% abundance by base pairs, while the 2 yeast species each had 2% abundance by base pairs. Further, we set each simulated dataset at 1 billion base pairs, meaning 120 million base pairs came from each of the bacterial genomes and 20 million base pairs from each of the yeast genomes. The average length and stddev of reads were 7,000 and 13,000 respectively (i.e., we provided the switch `--length 7000,13000` for all datasets). We did not simulate junk reads, random reads, or chimeras (i.e., we provided switches `--junk_reads 0`, `--random_reads 0`, and `--chimeras 0` for all datasets). Additionally, we used the seed 42 (`--seed 42`). Finally, we set the `--identity` switch to `95,99,2.5` for the 5% error rate reads (meaning the reads had an average identity of 95% with the reference sequences, a maximum identity of 99%, and a stddev of 2.5%), `90,99,2.5` for the 10% error rate reads, and `85,99,2.5` for the 15% error rate reads.

Much like the real reads, we classified the simulated reads with maximum read length cutoffs (ℓ values, see Section 3.1) of 180bp, 360bp, 720bp, 1440bp, and the full reads. Figures S4-S9 show the species-level accuracy results.

S4 Additional Tables and Figures

Throughput tables for the Bench and Bmock12-10kb reads are shown in Table S1 and Table S2 respectively. The supplementary Excel files provide the raw numbers to calculate recall, precision, accuracy, etc. for all experiments.

References

[1] R.R. Wick. Badread: simulation of error-prone long reads. *Journal of Open Source Software*, 4(36):1316, 2019.

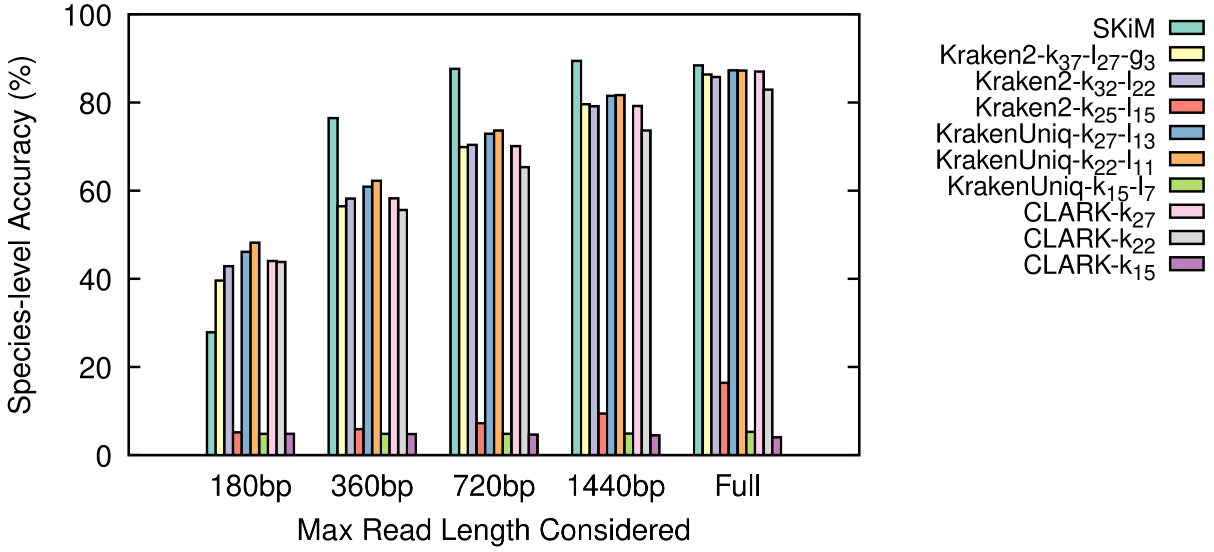

Figure S1: Species-level accuracy of other classifier parameters on the Even reads.

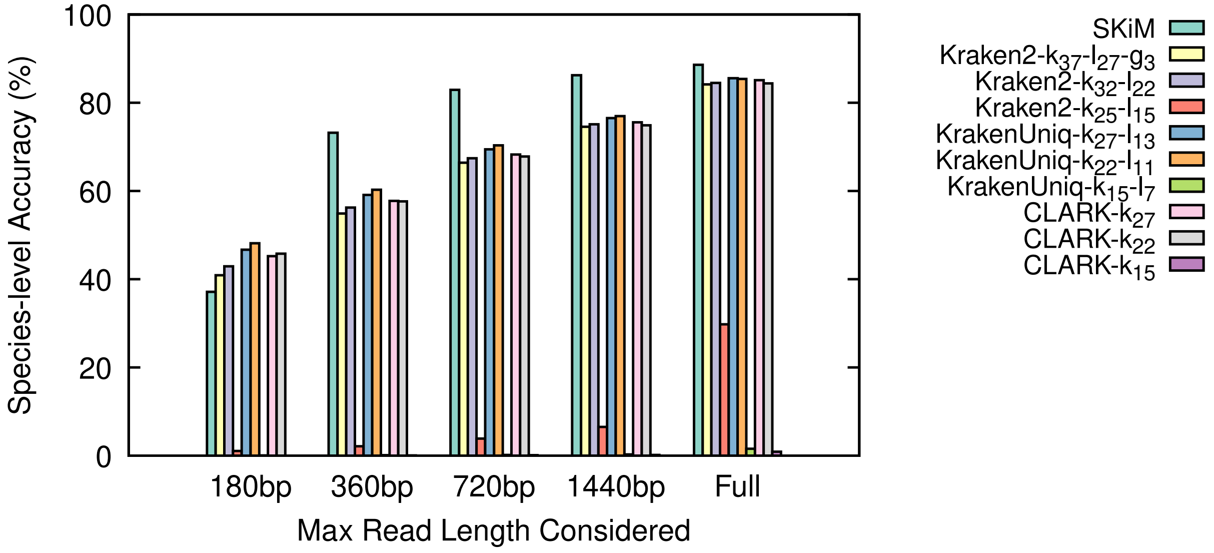

Figure S2: Species-level accuracy of other classifier parameters on the Bench reads.

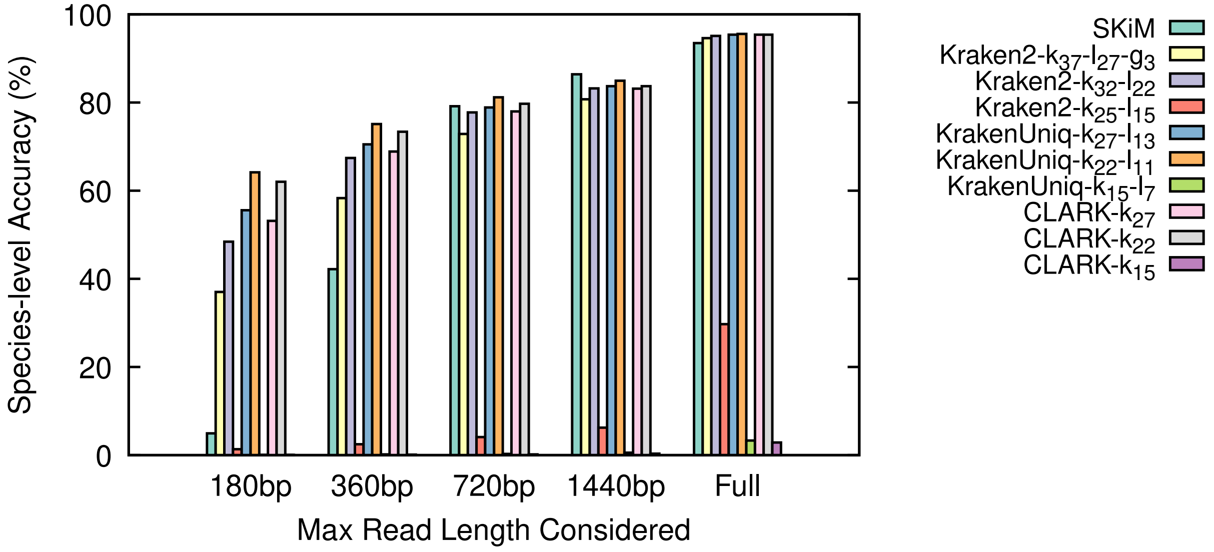

Figure S3: Species-level accuracy of other classifier parameters on the BMock12-10kb reads.

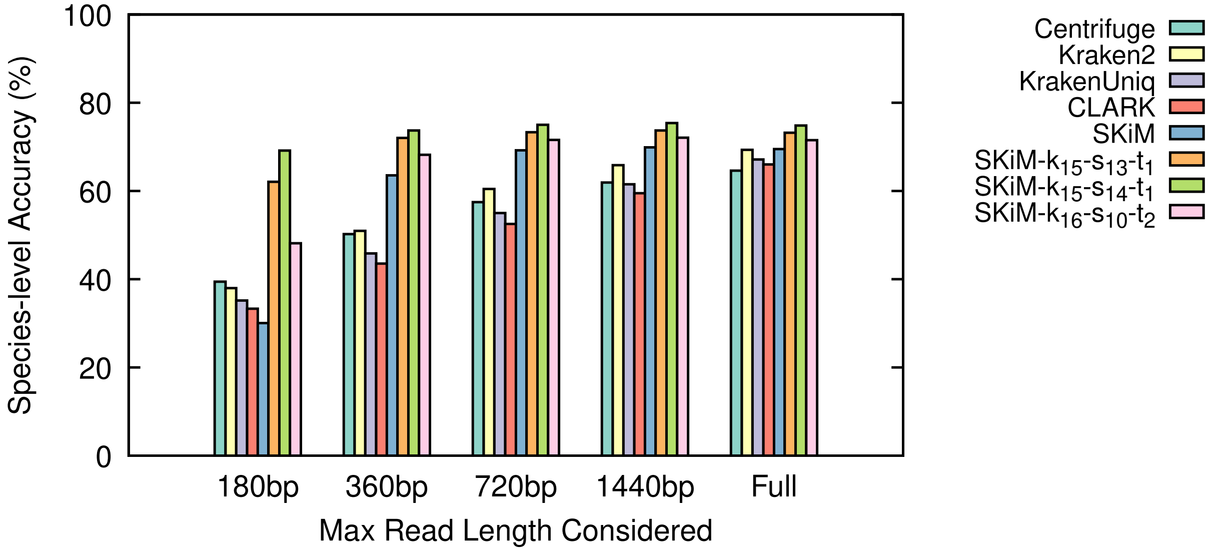

Figure S4: Species-level classifier accuracy on the simulated reads with 95% identity.

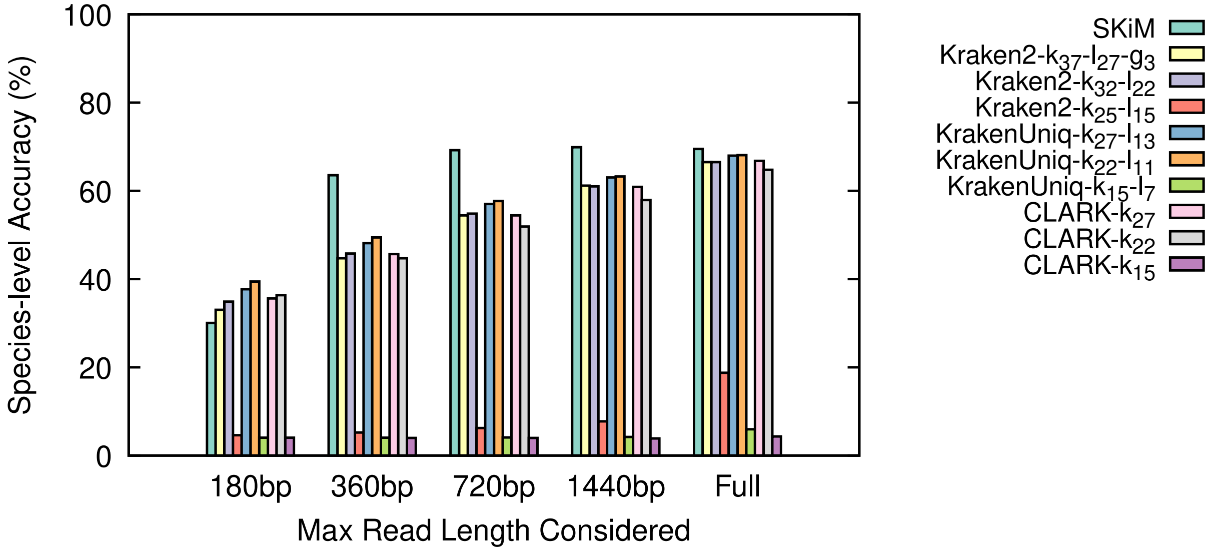

Figure S5: Species-level accuracy of other classifier parameters on the simulated reads with 95% identity.

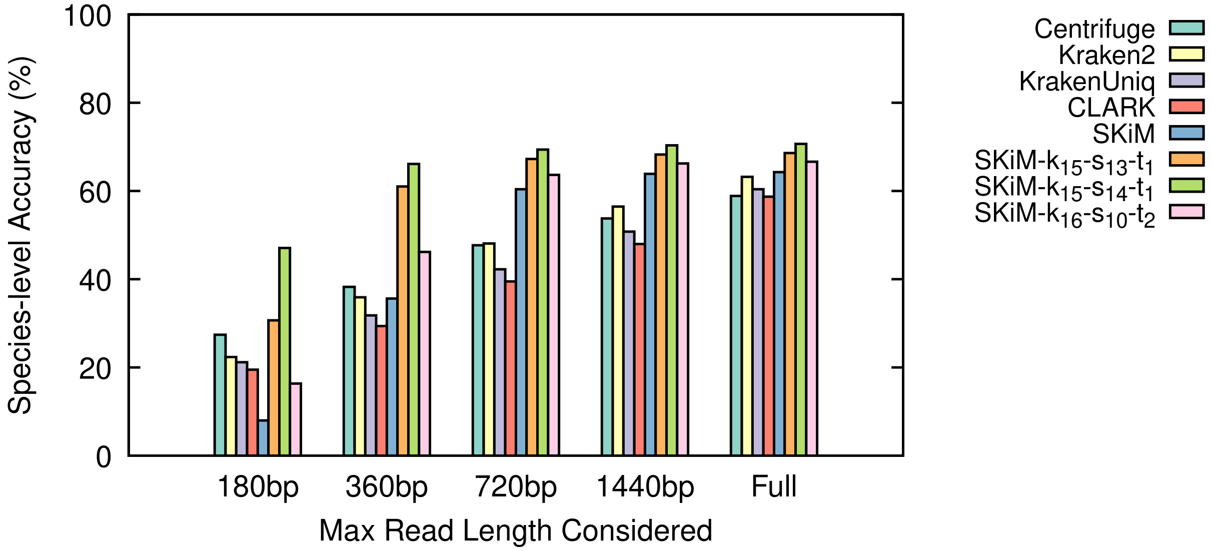

Figure S6: Species-level classifier accuracy on the simulated reads with 90% identity.

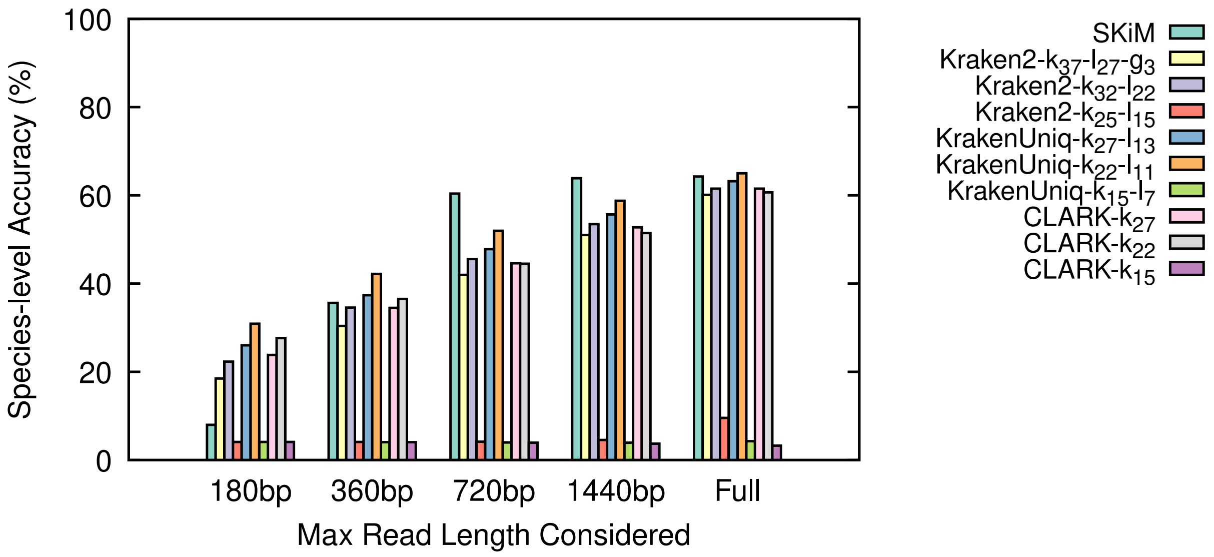

Figure S7: Species-level accuracy of other classifier parameters on the simulated reads with 90% identity.

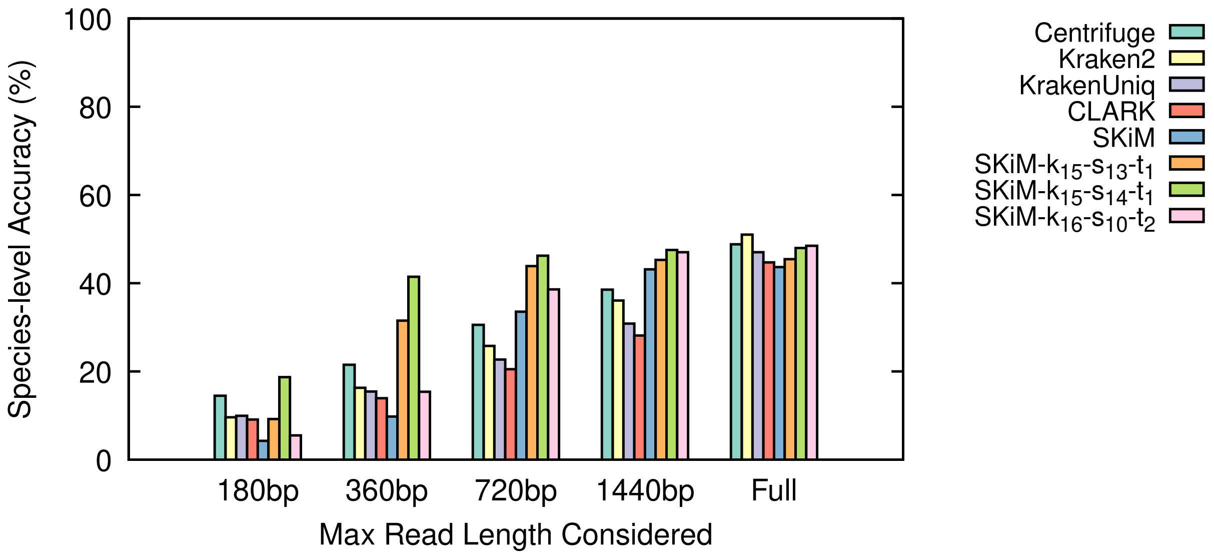

Figure S8: Species-level classifier accuracy on the simulated reads with 85% identity.

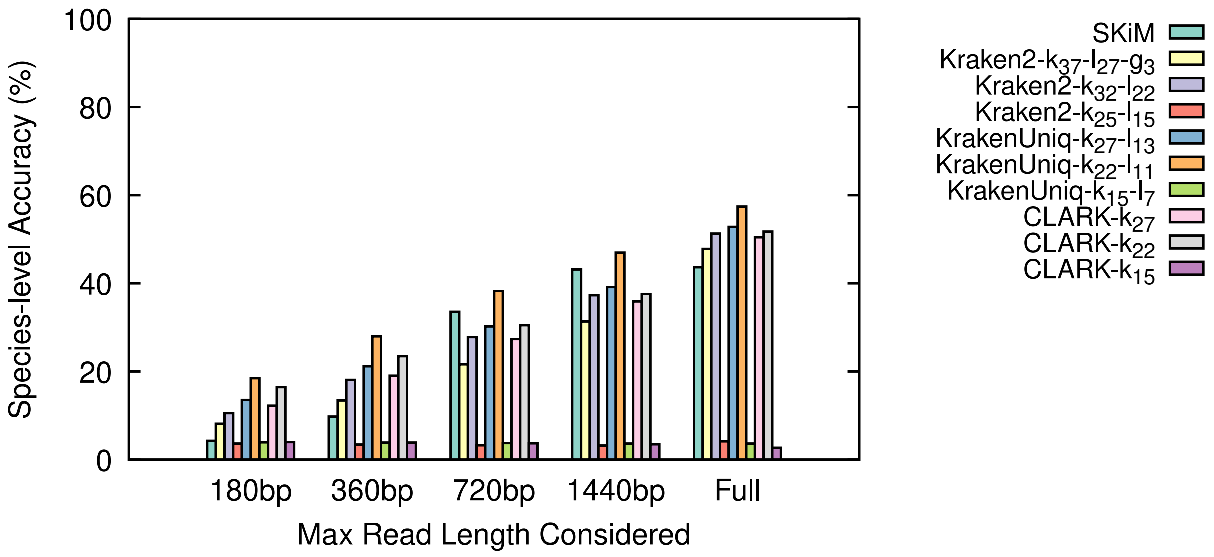

Figure S9: Species-level accuracy of other classifier parameters on the simulated reads with 85% identity.

Table S1: Classifier throughput on the Bench reads.

	**Max Read Length Considered**				
	**180bp (Mbp/s)**	**360bp (Mbp/s)**	**720bp (Mbp/s)**	**1440bp (Mbp/s)**	**Full (Mbp/s)**
Centrifuge	11.1	14.1	16.0	14.3	14.9
Kraken2	94.1	87.7	97.0	97.5	98.1
Kraken2-k_{37}-l_{27}-g_3	132.2	130.1	134.9	137.6	139.3
Kraken2-k_{32}-l_{22}	116.2	125.5	129.4	126.1	131.4
KrakenUniq	14.4	15.5	17.0	2.8	19.3
KrakenUniq-k_{27}-l_{13}	8.4	9.1	9.7	10.0	10.3
KrakenUniq-k_{22}-l_{11}	5.8	6.1	6.3	6.4	6.4
CLARK	26.6	25.3	24.7	24.5	24.7
CLARK-k_{27}	25.2	24.4	24.1	24.0	24.4
CLARK-k_{22}	24.2	23.5	23.3	23.1	23.6
SKiM	9.8	13.0	17.5	19.9	26.0
SKiM-k_{15}-s_{13}-t_1	7.0	9.3	10.5	12.7	15.0
SKiM-k_{15}-s_{14}-t_1	5.7	6.5	8.2	9.4	10.6
SKiM-k_{16}-s_{10}-t_2	14.4	19.5	24.7	27.9	38.2

Table S2: Classifier throughput on the Bmock12-10kb reads.

	Max Read Length Considered				
	**180bp (Mbp/s)**	**360bp (Mbp/s)**	**720bp (Mbp/s)**	**1440bp (Mbp/s)**	**Full (Mbp/s)**
Centrifuge	16.9	33.7	33.6	29.7	22.0
Kraken2	89.9	99.7	97.9	99.6	101.7
Kraken2-k_{37}-l_{27}-g_3	112.2	124.9	129.0	143.5	147.0
Kraken2-k_{32}-l_{22}	106.6	119.6	122.6	127.5	133.0
KrakenUniq	11.4	12.8	14.3	16.4	20.1
KrakenUniq-k_{27}-l_{13}	6.6	7.7	8.7	9.4	10.3
KrakenUniq-k_{22}-l_{11}	4.7	5.5	5.9	6.2	6.4
CLARK	26.9	25.1	24.2	23.9	24.4
CLARK-k_{27}	25.9	24.4	23.7	23.8	24.2
CLARK-k_{22}	24.7	23.8	23.4	23.2	23.9
SKiM	9.8	13.0	17.5	19.9	26.0
SkiM-k_{15}-s_{13}-t_1	7.0	9.3	10.5	12.7	15.0
SkiM-k_{15}-s_{14}-t_1	5.7	6.5	8.2	9.4	10.6
SkiM-k_{16}-s_{10}-t_2	14.4	19.5	24.7	27.9	38.2
